# *N*-Methylparoxetine Blocked Autophagic Flux and Induced Apoptosis by Activating ROS-MAPK Pathway in Non-Small Cell Lung Cancer Cells

**DOI:** 10.3390/ijms20143415

**Published:** 2019-07-11

**Authors:** Kun Wang, Bonan Chen, Ting Yin, Yujuan Zhan, Yuhua Lu, Yilin Zhang, Jiawei Chen, Weijie Wu, Shikun Zhou, Wenli Mao, Yuhui Tan, Biaoyan Du, Xiaodong Liu, Hiuting Idy HO, Jianyong Xiao

**Affiliations:** 1 Research Center of Integrative Medicine, School of Basic Medical Sciences, Guangzhou University of Chinese Medicine, Guangzhou 510006, China; 2 Department of Pathology, Guangzhou University of Chinese Medicine, Guangzhou 510006, China; 3 Department of Biochemistry, Guangzhou University of Chinese Medicine, Guangzhou 510006, China; 4 Department of Anaesthesia and Intensive Care, The Chinese University of Hong Kong, Hong Kong SAR 999077, China

**Keywords:** *N*-Methylparoxetine, NSCLC, autophagy inhibition, ROS, MAPK, apoptosis

## Abstract

The main mechanistic function of most chemotherapeutic drugs is mediated by inducing mitochondria-dependent apoptosis. Tumor cells usually respond to upregulate autophagy to eliminate impaired mitochondria for survival. Hypothetically, inhibiting autophagy might promote mitochondria-dependent apoptosis, thus enhancing the efficacy of chemotherapeutic therapies. We previously identified *N*-methylparoxetine (NMP) as an inducer of mitochondrial fragmentation with subsequent apoptosis in non-small cell lung cancer (NSCLC) cells. We discovered that ROS was accumulated in NMP-treated NSCLC cells, followed by c-Jun *N*-terminal kinase (JNK) and p38 MAP kinase (p38) activation. This was reversed by the application of a reactive oxygen species (ROS) scavenger, *N*-acetylcysteine (NAC), leading to a reduction in apoptosis. Our data suggested that NMP induced apoptosis in NSCLC cells by activating mitogen-activated protein kinase (MAPK) pathway. We further speculated that the remarkable increase of ROS in NMP-treated NSCLC cells might result from an inhibition of autophagy. Our current data confirmed that NMP blocked autophagy flux at late stage wherein lysosomal acidification was inhibited. Taken together, this study demonstrated that NMP could exert dual apoptotic functions—mitochondria impairment and, concomitantly, autophagy inhibition. NMP-related excessive ROS accumulation induced apoptosis by activating the MAPK pathway in NSCLC cells.

## 1. Introduction

Lung cancer is the leading cause of cancer death globally, representing 18% of all cancer deaths [1]. Non-small cell lung cancer (NSCLC) is the most prevalent subtype of all lung cancers (approximately 80–85%) [2]. Adjuvant chemotherapy after surgical resection is the prioritized strategy against NSCLC to prolong survival. Although the investigation and development of targeted drugs and immunotherapy gained much progress in cancer therapy, the five-year survival rate is only 16%, indicating a poor outcome of prognosis [3].

Activation of apoptosis is one of the major mechanisms of chemotherapeutic actions. As mitochondria play a pivotal role on triggering apoptosis [4], currently many clinically-used anti-tumor drugs, including the first-line therapies such as cisplatin and paclitaxel, exert a killing effect on cancer cells at least in part by mitochondrial apoptotic priming, or direct impairment of mitochondrial respiratory chain complexes. With this strategy, high doses of chemotherapy are normally required for an effective anti-tumor effect. However, treatment with high dosages of non-targeted drugs is indeed a double-edged sword that the cytotoxicity affects tumor and healthy cells simultaneously.

It is well known that mitochondria in tumor cells are vulnerable due to its frequent membrane “fission”, and as a result generates excessive reactive oxygen species (ROS) like hydrogen peroxide (H_2_O_2_)—the predominant one, superoxide anions (O_2_^−^), and hydroxyl free radicals (HO) [5,6]. When ROS overwhelms the cellular antioxidant defense system, the MAPK signaling pathways are activated and eventually result in the programmed cell death [7]. To reduce oxidative damage and maintain cellular homeostasis, tumor cells tend to upregulate autophagy to eliminate defective mitochondria [8,9]. Autophagy acts as a conserved degradative process of misfolded proteins and damaged organelles. In this process, autophagic cargo is engulfed by autophagosome, which is then fused with lysosome to form autolysosome. Subsequently, the cargo is broken down by various cathepsins in the autolysosome for molecules recycling. This entire catabolic process of autophagy, also known as autophagic flux, is crucial for maintaining cellular homeostasis and organelle quality control [10].

**Figure 1:**
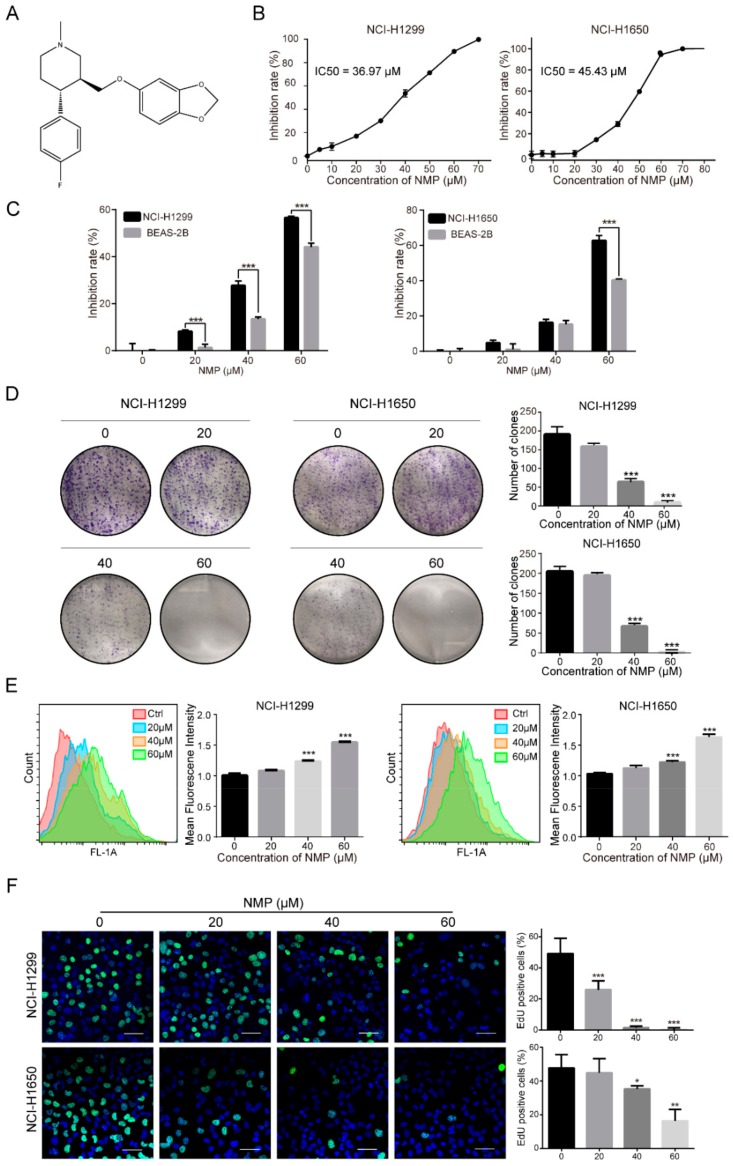
NMP inhibited human NSCLC cell proliferation. (**A**) Molecular structure of NMP. (**B**) Inhibition rates of proliferation in NMP-treated NCI-H1299 and NCI-H1650 cells (24 h) quantified by CCK-8 viability assay. Median inhibitory concentrations (IC50) were estimated by log(inhibitor) vs. normalized response of non-linear regression analysis. (**C**) Inhibition rates of proliferation in NMP-treated NCI-H1299, NCI-H1650 and BEAS-2B cells (24 h) quantified by CCK-8 viability assay. (**D**) Colony formation assay of NCI-H1299 and NCI-H1650 cells, treated with serial concentrations of NMP for 7 days. (**E**) Flow cytometry analyses of 24 h NMP (0–60 μM) treatment of CFDA-SE-labelled NCI-H1299 and NCI-H1650 cells (**F**) Fluorescence micrographs of NMP (0–60 μM, 24 h)-treated NCI-H1299 and NCI-H1650 cells with EdU incorporation. *Green*, EdU-positive cells; *blue*, Hoechst 33342 for nuclear staining. Scale bar, 20 μm. Error bars, means ± S.D. of three independent experiments; * *p* < 0.05, ** *p* < 0.01,*** *p* < 0.001, compared to the control group.

Recently, we screened the small molecule chemical library presented by Gu et al. [11] and discovered that *N*-methylparoxetine (NMP) was able to inhibit autophagy flux in NSCLC cells (data not shown). Hypothetically, inhibiting autophagy of cancer cells may undermine cellular redox state by excessive ROS accumulation and, consequently, apoptosis induction. Through indirect mitochondria impairment, NMP was expected to exert more striking killing effects in NSCLC cells with relatively low dosage. NMP is the precursor of paroxetine, a commonly used anti-depressant drug. Several studies have reported that paroxetine can exert a good anticancer activity in human cancer cells, including colon, oral, and breast cancer cells [12,13,14]. The structural resemblance of NMP and paroxetine implies that they may have similar physical, chemical, pharmacological, and adsorption, distribution, metabolism, excretion, and toxicological (ADMET) properties, with predictable clinical application potential (Figure 1A) [15]. 

In the present study, the anti-cancer effect of NMP on NSCLC cells was evaluated. Further, we revealed underlining mechanisms whereby NMP suppressed autophagy and induced apoptosis. The possible relationship between autophagy inhibition and apoptosis through ROS-MAPK signaling pathways was also examined.

## 2. Results

### 2.1. NMP Inhibited NSCLC Cells Proliferation

Firstly, we inspected the dose-dependent effect of NMP on NSCLC cell proliferation with CCK-8 assay. The IC50 for NCI-H1299 cells and NCI-H1650 cells were 36.97 μM and 45.43 μM, respectively (Figure 1B). Exposure to 60 μM or higher concentrations of NMP nearly inhibited all tumor cell growth, indicating its remarkable anti-proliferation activity. In addition, the effect of NMP on NSCLC cell lines and a normal lung epithelial cell line (BEAS-2B) was also compared, and NMP showed a higher inhibitory ability on NSCLC cells (Figure 1C). Furthermore, our colony formation assay showed that the numbers of colonies of NMP-pretreated NSCLC cells decreased in a dose-dependent manner. Only a few colonies were detected when either cell lines were treated with 60 μM NMP (Figure 1D). To determine the effect of NMP on cell division, NSCLC cells were labeled with CFDA-SE which can be equally distributed to daughter cells, leading to a decreased fluorescence intensity in proliferating cells. Following NMP treatment, increased fluorescent intensities in NSCLC cells was observed (Figure 1E). This indicated that NMP inhibited cell division. In addition, we performed 5-ethynyl-2-deoxyuridine (EdU) incorporation assay, which has been commonly used to indicate DNA synthesis, to confirm the effects of NMP on cell proliferation. The number of EdU-positive cells was decreased in NMP-treated group compared with the control group (Figure 1F). Altogether, these data showed that NMP had a significant inhibitory effect on NSCLC cell proliferation.

### 2.2. NMP Induced Apoptosis in NSCLC Cells

**Figure 2:**
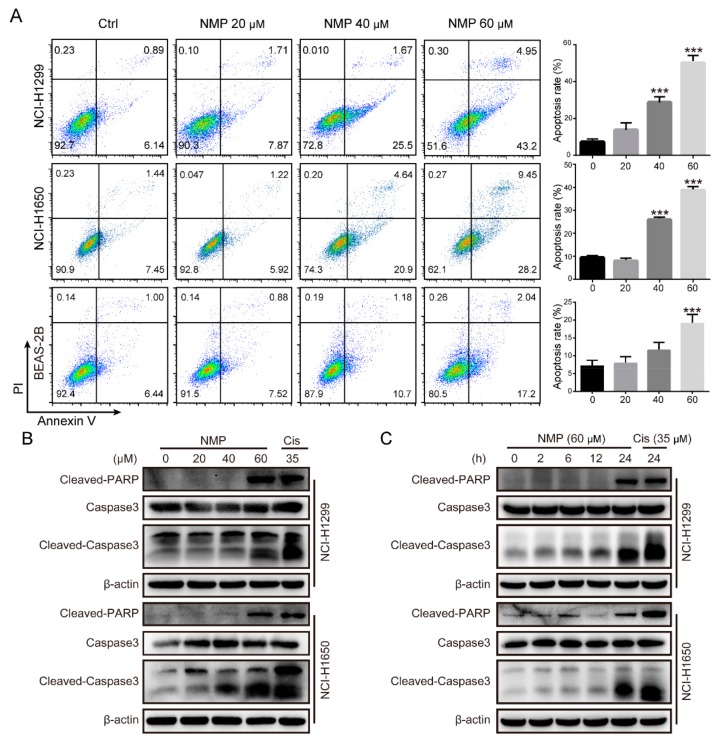
NMP induced apoptosis in NSCLC cells. (**A**) Flow cytometry analyses of NMP-treated NCI-H1299, NCI-H1650, and BEAS-2B cells that were subjected to PI/Annexin V staining assay for apoptosis detection. Error bars means ± S.D. of three independent experiments; *** *p* < 0.001, compared to the control group. (**B**,**C**) Western blots of whole cell lysates in NCI-H1299 and NCI-H1650 cells which were treated with NMP (60 µM) or cisplatin(Cis, 35 µM) at the indicated doses for 24 h (**B**) or for the indicated time courses (**C**).

NSCLC cells were double-stained with PI/Annexin V and analyzed by flow cytometry to access the apoptosis rate. As shown in Figure 2A, the percentage of PI/Annexin V double-positive cells increased in a dose-dependent manner after NMP treatment. In addition, NMP induces apoptosis in NSCLC cells more than in normal lung epithelial cells BEAS-2B. Consistent with these findings, western blot analysis showed that the apoptosis markers, cleaved-caspase 3 and cleaved-PARP, were upregulated following NMP treatment (Figure 2B,C). These results suggested that NMP induced apoptosis in NSCLC cells.

### 2.3. NMP Induced Apoptosis via a Mitochondria-Dependent Pathway

**Figure 3:**
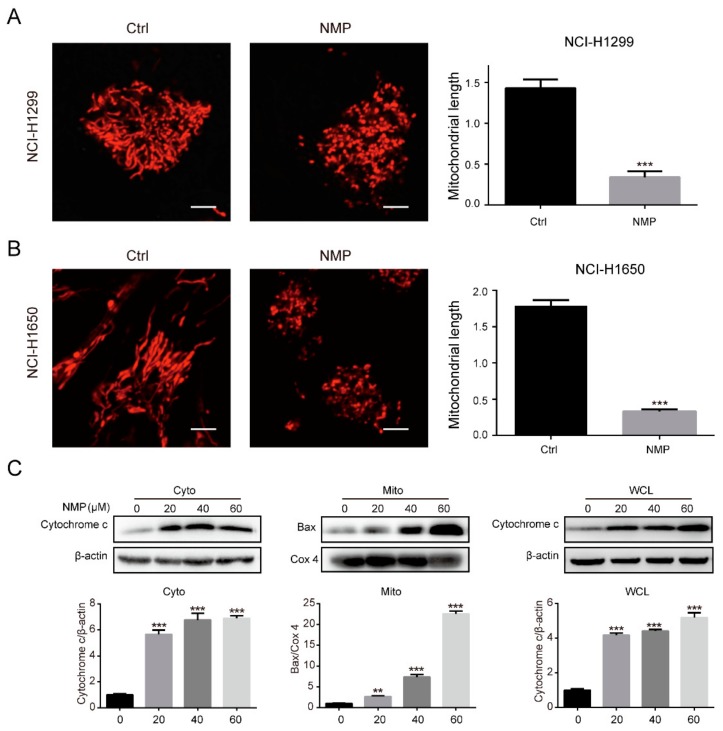
NMP induced apoptosis through a mitochondria-dependent pathway in NSCLC cell lines. (**A**,**B**) Fluorescence micrographs of mitochondria in a vehicle or 40 µM NMP-treated NCI-H1299 and NCI-H1650 cells with MitoTracker Red CMXRos staining. The length of mitochondria was quantified with ImageJ (US National Institutes of Health, Bethesda, MD, USA). Scale bar, 5 μm. Error bars mean ± S.D. of three independent experiments; *** *p* < 0.001, compared to the control group. (**C**) Western blot assay for mitochondria-dependent apoptosis of different cellular fractions obtained from NMP treated NCI-H1299 cells. The intensity of bands was quantified by using Gelpro32 Analyzer (Media Cybernetics, Inc., MD, USA). One-way analysis of variance (ANOVA), ** *p* < 0.01,*** *p* < 0.001, compared to the control group. *Cyto*, cytosolic fractions; *Mito*, mitochondrial fractions; *WCL*, whole cell lysates.

Mitochondria is a core player involved in the apoptosis induction. Hence, we asked if NMP induced apoptosis via the mitochondria-dependent pathway. Mitochondria morphological staining in NSCLC cells with MitoTracker Red CMXRos indicated that NMP treatment led to mitochondria fragmentation (Figure 3A,B). The fragmentation or impairment of mitochondria was confirmed by upregulation of the pore-forming protein, Bax, in mitochondrial fractions (Figure 3C). As mitochondria outer membrane permeability (MOMP) consequently caused cytochrome c release that subsequently activates intrinsic apoptotic cascade, we further evaluated the mitochondrial of cytochrome c by western blot. As shown in Figure 3C, cytochrome c levels were remarkably increased in both whole cell lysates and cytosolic fractions of NSCLC cells. These results suggested that NMP induced apoptosis through the mitochondria-dependent pathway in NSCLC cells.

### 2.4. NMP Induced ROS Accumulation and Activated MAPK Pathways

**Figure 4:**
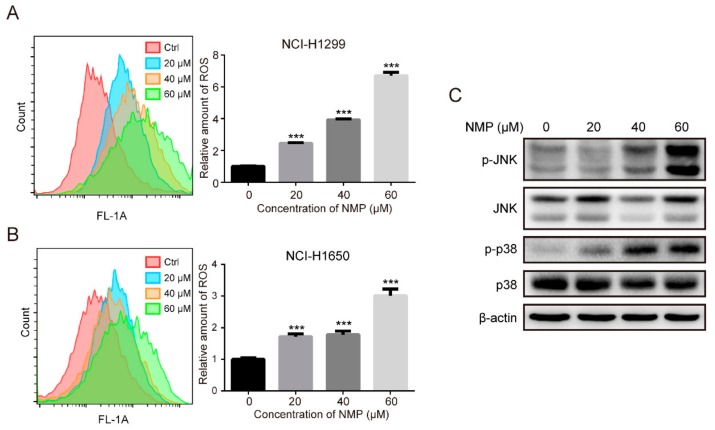
NMP induced ROS accumulation and activated MAPK pathways. (**A**,**B**) Flow cytometry analyses of intracellular ROS in NMP-treated NCI-H1299 and NCI-H1650 labeled with green DCFDA fluorescent dye. Error bars mean ± S.D. of three independent experiments; *** *p* < 0.001, compared to the control group. (**C**) Western blot assay for MAPK pathways in NMP-treated NCI-H1299 cells.

ROS is the main by-product generated by damaged mitochondria [6]. Therefore, we analyzed the amount of intracellular ROS in NSCLC cells probed by 2′,7′-dichlorofluorescein diacetate (DCFDA) with flow cytometry. NMP induced the accumulation of ROS in a dose-dependent fashion (Figure 4A,B). Excessive ROS is well known to activate MAPK pathways in which JNK and p38 are the key kinases mediating apoptosis. Phosphorylated-p38 and -JNK (active forms) were significantly upregulated after NMP treatment (Figure 4C), suggesting that NMP-induced apoptosis was MAPK pathway-dependent.

### 2.5. ROS Clearance Reversed the Activation of JNK/p38 and Attenuated NMP-Induced Apoptosis

**Figure 5:**
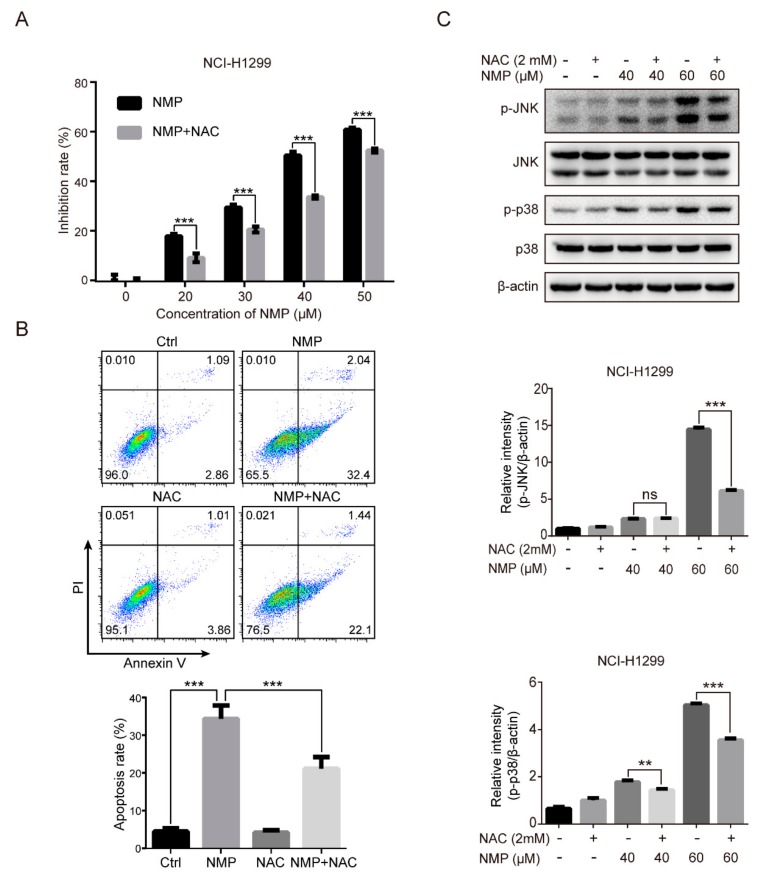
ROS clearance reversed JNK/p38 activation and attenuated NMP-induced apoptosis. (**A**) CCK-8 viability assay of NCI-H1299 cells treated with different concentrations of NMP with or without NAC (2 mM) for 24 h. (**B**) Flow cytometry analyses of the treated cells with Annexin V/PI labeling. (**C**) Western blot analysis for MAPK pathways of the treated cells. Error bars, means ± S.D. of three independent experiments; ** *p* < 0.01; *** *p* < 0.001, compared to the control group.

To determine the key roles of ROS in inhibiting cell proliferation and inducing apoptosis, cells were treated with *N*-acetylcysteine (NAC, a ROS scavenger) to reduce intracellular ROS, followed by CCK-8 assay and PI/Annexin V staining. Combined treatment with NMP and NAC led to significantly decreased cell proliferation inhibition rate in NSCLC cells compared with NMP treatment alone (Figure 5A). The apoptotic rate in NMP group (34.33% ± 3.53%) was decreased to 21.11% ± 3.10% in the combined treatment group (Figure 5B). These data indicated that NAC significantly attenuated NMP-induced-proliferative inhibition and apoptosis in NSCLC cells. In parallel, Western blot analysis showed that NAC treatment downregulated the phosphorylation forms of JNK and p38 in NMP-treated cells (Figure 5C), suggesting ROS clearance reversed NMP-induced JNK and p38 activation. Taken together, these results indicated that NMP induced apoptosis in NSCLC cells through ROS-MAPK pathways.

### 2.6. NMP Induced the Accumulation of Autophagosomes in NSCLC Cells

**Figure 6:**
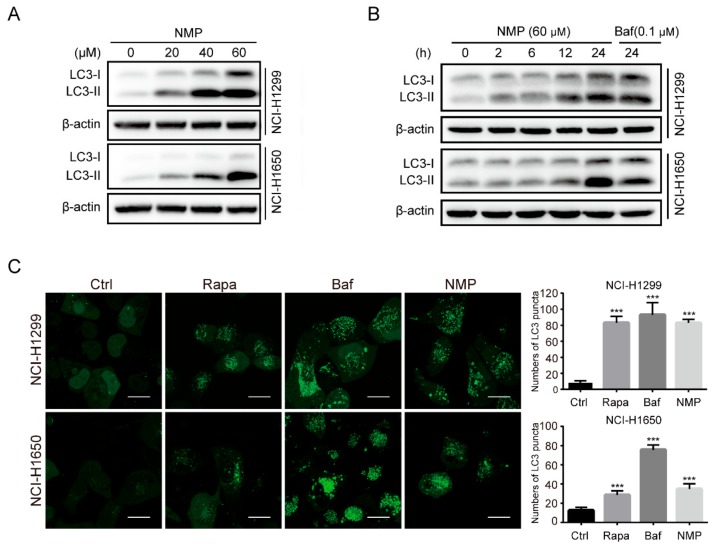
NMP induced the accumulation of autophagosomes in NSCLC Cells. (**A**,**B**) Western blot analyses for LC3II of dose-dependent (24 h treatment, (**A**)) or time-dependent responses (**B**) in NCI-H1299 and NCI-H1650 cells treated with NMP (60 µM) or bafilomycin A1 (Baf, 0.1 µM). (**C**) Fluorescence micrographs of LC3-stably-expressed NCI-H1299 and NCI-H1650 cells treated with vehicle, rapamycin (Rapa, 0.5 µM), bafilomycin A1 (Baf, 0.1 µM) or NMP (40 µM). Scale bar, 20 μm. Error bars mean ± S.D. of three independent experiments; *** *p* < 0.001, compared to the control group.

Although NMP was able to induce mitochondria fragmentation in NSCLC cells, the damaged mitochondria could be eliminated by autophagy which is usually upregulated in cancer cells to compensate for excessive ROS accumulation. It is possible that the remarkable increase of ROS in NMP-treated NSCLC cells may result from the blockade of autophagic flux. We examined LC3-II level, a canonical marker of autophagosome, in NSCLC cells following NMP or bafilomycin A1 (Baf, positive control) treatment. Western blot analyses showed that NMP treatment upregulated LC3-II in both concentration- and time-dependent manners (Figure 6A,B). In addition, NMP markedly increased GFP-LC3 puncta formation in NSCLC cells with GFP-tagged LC3 transfection, similar to the positive control groups (Rapa, rapamycin, an autophagy inducer; Baf, an autophagy suppressor blocking late autophagic flux, Figure 6C). These data suggested that NMP treatment induced the accumulation of autophagosomes in NSCLC cells.

### 2.7. NMP Impeded the Late-Staged Autophagic Flux in NSCLC Cells

**Figure 7:**
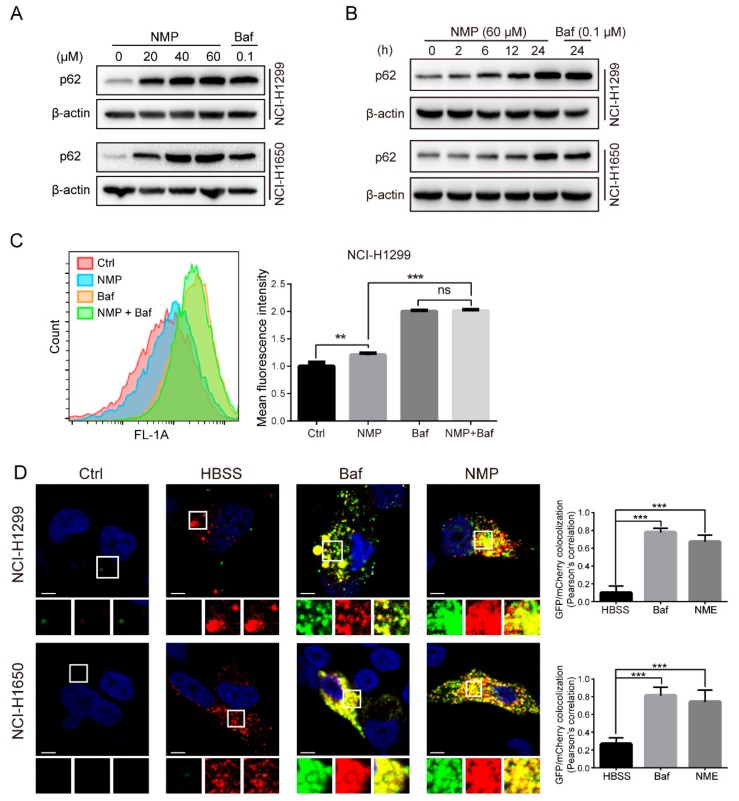
NMP impaired NSCLC cell late-staged autophagic flux. (**A,B**) Western blot analysis for p62 of NMP- of Baf-treated NCI-H1299 and NCI-H1650 cells at the indicated doses for 24 h (**A**), or 60 µM NMP- or 0.1 µM Baf-treated cells for the indicated time courses (B). (**C**) Flow cytometry analyses of GFP-LC3 mean fluorescence intensities in NCI-H1299 cells treated with NMP (40 µM), Baf (0.1 µM) or in combination. (**D**) *Left*, typical fluorescence micrographs of mCherry-GFP-LC3-expressing NCI-H1299 or NCI-H1650 cells treated with vehicle, NMP (40 µM), or Baf (0.1 µM) for 24 h; or HBSS for 6 h. Scale bar, 5 μm. *Right*, Pearson’s correlation analyses of GFP/mCherry colocalization. Error bars, means ± S.D. of three independent experiments; ** *p* < 0.01, ***p < 0.001, compared to the control group.

Autophagic flux involved orchestrated autophagosome initiation, lysosomal fusion, and degradation. Elevated autophagosomes formation may represent the promotion of autophagosome initiation (induction of early-staged autophagic flux) or the blockade of autophagosome degradation (inhibition of late-staged autophagic flux) [16,17]. Our data showed that NMP upregulated p62, a commonly used indicator of autophagic flux inhibition, time- and dose-dependently in NSCLC cells (Figure 7A,B), suggesting that NMP was probably an autophagy inhibitor. We further determined which stages were blocked by NMP. Baf blocks late autophagic flux through inhibiting V-ATPase localized in the lysosomal membrane. Our flow cytometry analyses showed that NSCLC cells treated with NMP expressed similar mean GFP-LC3 fluorescence intensities to Baf treatment group (Figure 7C), suggesting that NMP inhibited late autophagic flux. In addition, the blocked stages of autophagy by NMP were tested with mCherry-GFP-LC3 transfected NSCLC cells. The fluorescent signal of GFP is quenched in an acidic environment, while mCherry exhibits more stable fluorescence in acidic compartments. As shown in Figure 7D, NMP or Baf treatment resulted in a larger portion of yellow puncta, labeling autophagosomes (merge of mCherry and GFP), compared with HBSS group in which mCherry signals, labeling autolysosome, were predominant. Pearson’s correlation statistics were exploited to analyze the colocalization of GFP-LC3/mCherry fluorescence. Either NMP- or Baf-treated cells had significantly higher colocalization ratios than HBSS-treated cells (Figure 7D, right panel). These data suggested that NMP inhibited late autophagy flux, resulting in marked autophagosome accumulation.

### 2.8. NMP Altered the Lysosomal pH and Inhibited Lysosomal Cathepsins Maturation

**Figure 8:**
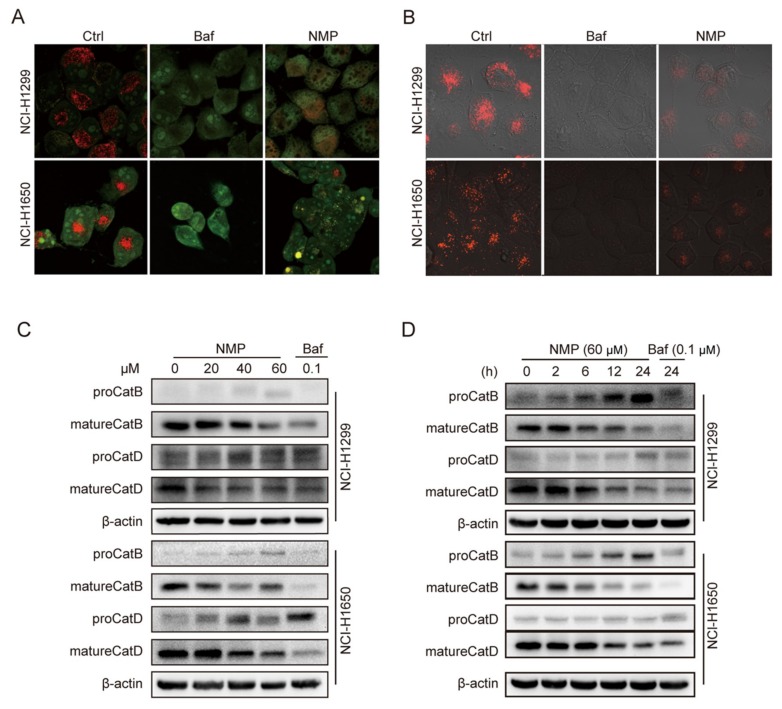
NMP altered lysosomal pH and inhibited lysosomal cathepsins maturation. (**A**,**B**) Typical fluorescence micrograph of vehicle-, Baf (0.1 µM)- or NMP (40 µM)-treated NCI-H1299 and NCI-H1650 cells subjected to pH-dependent fluorescent dyes acridine orange (AO) and Lysotracker Red DND-99 stainings to detect lysosomal acidification. Scale bar, 20 μm. (**C**,**D**) Western blot analysis for mature cathepsin B and D detection in treated cells in dose- (**C**) or time-dependent manners (**D**).

Subsequent to autolysosome formation, aberrant lysosomal pH could suppress the maturation of lysosomal cathepsin and slow down cargo degradation, consequently impede autophagic flux at a later stage [17]. To determine if NMP affects lysosomal acidification, the cells treated with vehicle, NMP or Baf, an established inhibitor of V-ATPase as a positive control [18], were stained with acridine orange (AO) or LysoTracker Red. Compared with the negative control, NMP markedly reduced red fluorescence of AO (Figure 8A) or LysoTracker Red (Figure 8B). In parallel, Western blot analysis showed that NMP reduced mature cathepsin B and D levels compared with negative control, in a dose- (Figure 8C) and time-dependent manner (Figure 8D). These data confirmed that NMP suppressed late-staged autophagy by altering lysosomal pH.

## 3. Discussion

**Figure 9:**
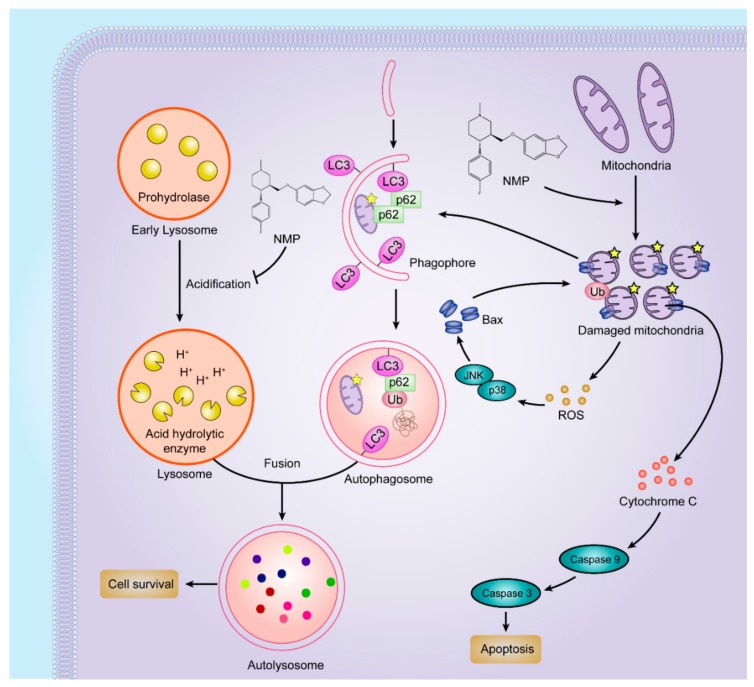
NMP induced apoptosis in NSCLC cells by dual pathways. An illustration demonstrating the summary of NMP actions on apoptosis in NSCLC cells. (**Left**) NMP inhibited late-staged autophagy flux by inhibiting acidification of early lysosome. (**Right**) NMP stimulated mitochondrial fission and fragmentation, leading to ROS and cytochrome C leakage. Cytochrome C is a risk factor to promote apoptosis. Excessive ROS accumulation further damages mitochondria, thus NMP could induce an effective apoptotic effect on tumor cells by promoting mitochondrial-dependent apoptosis and inhibiting autophagy in NSCLC cells in parallel.

In this study, we proposed that NMP was an effective inducer of apoptosis in NSCLC cells by dual pathways (Figure 9). On one hand, NMP stimulated mitochondrial fission and fragmentation. Fragmented mitochondria showed round morphology (i.e., depolarization) with MOMP leading to the leakage of ROS and apoptosis factors, such as cytochrome c. In favor of homeostasis maintenance, cells can eliminate impaired mitochondria to avoid excessive accumulation of ROS by autophagy (“self-eating”). However, exclusively striking mitochondria may not effectively kill cancer cells due to the concurrent upregulated autophagic pathway. Recently, it has been confirmed that inhibition of autophagy can be a new strategy to improve the efficacy of mitochondrial disrupting agents [19]. In this study, we demonstrated that NMP inhibited autophagy in NSCLC cells. NMP disrupted the ROS removal via blockade of the late-staged autophagy flux. As an excessive accumulation of ROS is a risk factor damaging mitochondria [20], NMP can exert more efficient apoptotic effects on cancer cells with this positive feedback loop from ROS to mitochondria. This reassures the future development of NMP as a promising drug for clinical chemotherapeutic application. 

To delineate the actions of NMP on autophagy, we firstly quantified autophagosome formation in NSCLC cells. Autophagosome, a double-membraned structure, was initiated from the endoplasmic reticulum. Cargos, such as unfolded proteins and damaged organelles, were mediated by receptors and capsuled in autophagosome, fused with lysosome to form, and degraded in autolysosome. Accordingly, autophagosome is a central structure during autophagosome flux. Secondly, we examined LC3-II expression. LC3-II, a well-established marker of autophagosome, is generated by cleavage of its precursor LC3 and conjugation to lipid phosphatidylethanolamine (PE) in the autophagosome, and eventually degraded alongside the cargo. Although the expression of LC3-II was upregulated in NSCLC cells following NMP treatment, the data did not indicate if NMP inhibited or promoted autophagy. Therefore, we then measured the expression levels of p62, which is responsible for recruiting ubiquitinated-substrate (i.e., the cargo) to the LC3-II autophagosomal receptor, preceding degradation. Autophagy-dependent pathway remains the prevailing degradation pathway of p62 although it can also be degraded by proteasome. The dose-dependent upregulation of p62 in our data suggested that NMP inhibited autophagy of NSCLC cells. Further, we demonstrated that NMP inhibited late staged autophagy flux. Specifically, we treated GFP-LC3-expressing NSCLC cells with NMP in combination with a widely accepted late autophagy inhibitor, bafilomycin A1 and quantified the GFP-LC3 signals. If NMP inhibited early autophagy, the combined treatment would lead to the low intensity of GFP-fluorescence. Our data showed a similar intensity of GFP-LC3 fluorescence intensities between the combined treatment and the bafilomycin A1-standalone group. These results suggested that NMP inhibited late autophagy as bafilomycin A1 did. In addition, NSCLC cells were transiently transfected with mCherry-GFP-LC3 constructs to trace autophagy flux. The tandem fluorescence system labels autophagosome with yellow fluorescence (i.e., merging of green and red) and autolysosome with a red signal due to quenching of GFP in an acidic environment. A large proportion of yellow fluorescence was detected in the NMP group, similar to that in bafilomycin A1 group. We speculated that NMP might suppress lysosomal acidification. This was confirmed by decreased AO and lysotracker stainings, and the impeded maturation of lysosomal cathepsins. Of note, here lies another uncertainty—the fusion of autophagosome and lysosome may be obstructed. To clarify the action of NMP on it, we specifically labeled lysosome in GFP-LC3-expressing NSCLC cells with LysoBrite^TM^ Red. In contrast to the positive control group treated with chloroquine (CQ) group, a fusion inhibitor, NMP group showed a larger percentage of colocalizing autophagosome and lysosome (yellow fluorescence, Supplementary Figure S1), suggesting treatment of NMP did not inhibit the fusion process to form autolysosome.

The accumulation of autophagosomes was reported to be stress leading to autophagic cell death [18,19]. To test whether NMP induced cell death is due to autophagic cell death, 3-methyladenine (3-MA), an inhibitor of early autophagy targeting PI3K (also termed Vps34), was used to block autophagosome formation [16]. The combination of NMP and 3-MA did not show any decreased, but indeed increased inhibition rate of lung cancer cells proliferation compared with the NMP treatment alone (Supplementary Figure S2A,B). This result was then confirmed by apoptotic analysis with PI/Annexin V staining (Supplementary Figure S2C). Since 3-MA treatment was not able to reverse the inhibitory effect of NMP on NSCLC cells proliferation, NMP-induced cell death could not be explained by autophagic cell death. 

Excessive ROS accumulation is well known to activate MAPK pathways, resulting in cell death. For example, palmitate could induce H9c2 cell death through activation of ROS/MAPK signaling pathways [21]. Also, isoliensinine could induce apoptosis in human breast cancer cells through ROS generation and p38/JNK activation [22]. Our data showed that NMP treatment resulted in a significant increase of intracellular ROS (Figure 4A,B) and activation of p38/JNK (Figure 4C). To test whether ROS is involved in regulating MAPK pathways, we treated NSCLC cells with a ROS scavenger, NAC, and found a significant reduction in NMP-induced apoptosis (Figure 5B) with abolished activation of p38 and JNK (Figure 5C). These data indicated that ROS might induce apoptosis by activating p38/JNK. 

Several lines of evidence have established that phosphorylation of Bcl-2 by p38 enhanced its degradation via a proteasome-dependent pathway. JNK pathway regulates mitochondrial apoptotic cell death via modulation of Bcl-2 and Bax protein expression [23,24,25,26]. However, our data showed that NMP did alter the expression of Bcl-2. JNK and p38 were reported to mediate the translocation of Bax from the cytoplasm to the mitochondrial outer membrane by Bax phosphorylation [27]. The Bax protein plays a crucial role in apoptosis induction through the mitochondria-dependent pathway that controls the release of cytochrome c, a well-known apoptotic factor. When the Bax protein is translocated to the mitochrondrial outer membrane, channels for cytochrome c release were formed [28]. In this study, we found that Bax was accumulated in the mitochrondria fraction while cytochrome c was markedly increased in the cytosolic fraction (Figure 3C). This suggested that NMP treatment induced Bax translocation to mitochondria, followed by cytochrome c release to cytosol. Collectively, we concluded that NMP induced mitochondria-dependent apoptosis in human NSCLC cells. This was mediated by p38 and JNK activation followed by Bax translocation.

## 4. Materials and Methods

### 4.1. Cell Culture and Drugs

Human NSCLC cell lines NCI-H1299 (ATCC^®^ CRL-5803™) and NCI-H1650 (ATCC^®^ CRL-5883™) were purchased from American Type Culture Collection (ATCC, Rockville, MD, USA). BEAS-2B, an immortalized human bronchial epithelial cell line, was purchased from iCell Bioscience, Inc. (iCell-h023, Shanghai, China). All cells were cultured in Dulbecco’s modified Eagle’s medium (DMEM) supplemented with 10% fetal bovine serum (FBS) and 100 U/mL penicillin/streptomycin at 37 °C in 5% CO_2_. All reagents for cell culture were purchased from Gibco Life Technologies (Grand Island, NY, USA) unless otherwise stated.

*N*-Methylparoxetine (NMP, M2645) was purchased from J and K Chemical (Beijing, China). Bafilomycin A1 (Baf, B1793) was purchased from Sigma Biotechnology (St. Louis, MO, USA). Rapamycin (Rapa, S1039) was purchased from Selleckchem (Houston, TX, USA).

### 4.2. Cell Viability Assay

Cells were seeded onto 96-well plates and cultured overnight before treatment with different compounds for 24 h. Cell proliferation was assessed using Cell Counting Kit-8 (CCK-8, CK04, Dojindo Laboratories, Kumamoto, Japan) according to the manufacturer’s instruction. Briefly, the medium of each well was removed before adding 100 µL of CCK-8 reagent (10-fold dilution in DMEM). The plates were incubated for 2 h at 37 °C before measuring absorbance at 450 nm.

### 4.3. Colony Formation Assay

Cells were seeded onto 6-well plates, cultured for two days and treated with drugs for three days. The medium was removed, followed by rinsing colonies with PBS and fixation with 4% paraformaldehyde (PFA) for 20 min. The colonies were then stained with crystal violet and photographed manually.

### 4.4. CFDA-SE Cell Tracking Assay

Cells were labeled with CFDA-SE Cell Proliferation Assay and Tracking Kit (C0051, Beyotime, Shanghai, China) and seeded in six-well plates with overnight incubation before drug treatment for 24 h. The cells were harvested and washed with PBS before resuspension in HBSS for fluorescence intensity quantification with a BD Accuri^TM^ C6 flow cytometer (BD Pharmingen, San Diego, CA, USA).

### 4.5. 5-Ethynyl-20-Deoxyuridine (EdU) Cell Proliferation Assay

Cells were seeded on a confocal dish and cultured overnight before treatment for 24 h. 5-Ethynyl-20-deoxyuridine agent (EdU, C0071S, Beyotime, Shanghai, China) was added to each dish and allowed 8 h for incorporation. The cells were fixed in 4% PFA for 20 min and washed three times with PBS containing 3% bovine serum albumin (BSA). The cells were incubated in PBS containing 0.3% TritonX-100 for 15 min, followed by BSA-containing PBS washes three times. The cells were stained with 5 μg/mL Hoechst 33342 at room temperature for 10 min, washed three times with PBS and mounted using Prolong Diamond DAPI (P36966, Karlsruhe, Germany). Images were captured using an LSM 800 confocal microscopy platform (Carl Zeiss, Jena, Germany).

### 4.6. Cell Apoptosis Detection

Cells were plated onto six-well plates overnight and treated for 24 h. Cell apoptosis detection was performed with FITC Annexin V Apoptosis Detection Kit (556547, BD Pharmingen, San Jose, CA, USA) according to manufacturer’s instruction. Briefly, treated cells were harvested and washed with PBS, followed by suspension in 600 μL annexin V binding buffer. Three microliters of annexin-V-FITC was added to each sample for 20 min at 37 °C in the dark. Then 5 μL PI was added for a further 5 min incubation. For each sample, 10,000 cells were analyzed using FL1 and FL3 channels with an Accuri C6 flow cytometer (BD Pharmingen, San Diego, CA, USA).

### 4.7. Cellular Fractionation Western Blot Analysis

Cellular fractions from treated cells were extracted with a mitochondrial protein extraction kit (BB-3171) and cytosolic protein extraction kit (BB-3113) from BestBio (Shanghai, China). Treated cells were harvested and washed twice with PBS. Five-hundred μL of mitochondrial or cytoplasmic protein reagent was added to each sample, followed by incubation on ice for 20 min. The samples were vortexed every 5 min. Mitochondrial protein was prepared by centrifugation at 11,000× g for 20 min while cytoplasmic protein was prepared by centrifugation at 16,000× g for 5 min. The mitochondrial protein was lysed in 1× loading buffer. The cytoplasmic protein was quantified using a BCA protein assay kit (MA0082, Meilunbio, Dalian, China). All proteins were stored at −80 °C.

### 4.8. Western Blot Analysis

The whole cell was lysed in 1× loading buffer. The protein samples were separated with 12% sodium dodecyl sulfate-polyacrylamide gel electrophoresis (SDS-PAGE) and transferred onto nitrocellulose membranes. The membranes were blocked in 5% skim milk powder in TBST for 2 h and incubated overnight with primary antibodies at 4 °C. The membrane was washed three times with TBST (0.05% Tween 20 in Tris-buffered saline) and incubated with secondary antibodies (diluted 1:4000). The immunoreactive bands were visualized with enhanced chemiluminescence (ECL) using an ECL detection system. The band densities were quantified using ImageJ (NIH).

Anti-LC3B antibody (12994S), anti-p62 antibody (5114), anti-PARP antibody (9542), anti-P-JNK antibody (9251), anti-JNK antibody (9252), anti-CytochromeC antibody (11940), and cleaved-caspase 3 antibody (9662) were from Cell Signaling Technology (Boston, MA, USA). CatD antibody (SC-13985) was from Santa Cruz Biotechnology (Dallas, TX, USA). Peroxidase-labeled antibodies anti-mouse IgG (AS003) and anti-rabbit IgG (AS014) were from ABclonal (Wuhan, China).

### 4.9. Plasmid Transfection Assay

Cells were seeded onto coverslips in 12-well plates and cultured for 24 h. GFP-LC3B or mCherry-GFP-LC3B plasmids (generous gifts from Professor William K. K. Wu, The Chinese University of Hong Kong) were transfected into cells using Lipofectamine 3000 strictly according to the manufacturer’s instructions (Invitrogen). After 6 h, the transfection medium was replaced with fresh culture medium and the cells were incubated for 24 h before drug treatment. The cells were fixed with 4% PFA for 20 min and washed three times with PBS. Five μg/mL Hoechst 33342 was added for 10 min staining at room temperature. Stained cells were mounted on glass slides using Prolong Diamond DAPI and subjected to confocal imaging.

### 4.10. AO Staining

Cells were seeded onto coverslips in 12-well plates and incubated for 24 h before drug treatment. The medium was removed and the cells were washed three times with PBS. Each sample was incubated with 5 μg/mL Acridine Orange (AO, A6014, Sigma Biotechnology, St. Louis, MO, USA) at 37 °C with 5% CO_2_ for 20 min and washed three times with PBS. Images were obtained with a laser confocal scanning microscope equipped with an argon laser (excitation wavelength: 488 nm) and a 63× objective lens. In the lysosomal compartments, AO produces red fluorescence (620 nm long pass emission filter) and green fluorescence (emission range: 520–560 nm) in the cytosol and nuclear compartments. The red and green intensities’ ratios in non-nuclear cellular compartments were analyzed using ImageJ.

### 4.11. LysoTracker Red Staining

Cells were seeded onto coverslips in 12-well plates and cultured for 24 h before drug treatment. The medium was removed and the cells were washed three times with PBS. Each sample was stained with LysoTracker Red DND-99 (L7528, Invitrogen, Carlsbad, CA, USA) for 20 min. Then the samples were washed three times with PBS and imaged under the laser scanning confocal microscope equipped with an argon laser (excitation wavelength: 555 nm) and a 63× objective lens.

### 4.12. MitoTracker Red CMXRos Staining

Cells were seeded onto coverslips in 12-well plates and cultured for 24 h before drug treatment. The medium was removed and the cells were washed three times with PBS. Each sample was stained with MitoTracker Red CMXRos (9082) from Cell Signaling Technology, Boston, MA, USA) for 20 min and washed three times with PBS. Images were obtained with a laser scanning confocal microscope equipped with an argon laser (excitation wavelength: 555 nm) and a 63× objective lens.

### 4.13. Intracellular ROS Measurement

Cells were seeded onto 6-well plates and cultured for 24 h before drug treatment. The cells were harvested and washed with PBS, followed by staining with 10 μM DCFH-DA (D399) from Thermo Fisher Scientific (Waltham, MA, USA) for 15 min at 37 °C in dark. Each sample was washed with PBS and resuspended in HBSS. From each sample, 10,000 cells were analyzed using the FL1 channel with a BD Accuri C6 flow cytometer.

### 4.14. Statistical Analysis

All experiments were repeated at least three times. The statistics were evaluated using one-way analysis of variance (ANOVA) and multiple comparisons. Level of significance was set at *** *p* < 0.001; ** *p* < 0.01; and * *p* < 0.05.

## 5. Conclusions

In this study, we identified NMP as an inducer of mitochondrial fragmentation with subsequent apoptosis in NSCLC cells. Then we discovered that ROS was accumulated in NMP-treated NSCLC cells, followed by c-Jun *N*-terminal kinase (JNK) and p38 MAP kinase (p38) activation, and this was reversed by NAC, leading to a reduction in apoptosis. Our data suggested that NMP induced apoptosis in NSCLC cells by activating the mitogen-activated protein kinase (MAPK) pathway. Additionally, our current data showed that NMP blocked autophagy flux at the late stage wherein lysosomal acidification was inhibited. Collectively, this study demonstrated that NMP could exert dual apoptotic functions—mitochondria impairment and, concomitantly, autophagy inhibition.

## Supplementary Materials

The following are available online at https://www.mdpi.com/1422-0067/20/14/3415/s1.

## Abbreviations

NMP	*N*-Methylparoxetine
NSCLC	Non-small cell lung cancer
NAC	*N*-acetylcysteine
ROS	Reactive oxygen species
MAPK	mitogen-activated protein kinase
JNK	c-Jun *N*-terminal kinase
p38	p38 MAP kinase
ADMET	Adsorption, distribution, metabolism, excretion, and toxicity
IC50	Median inhibitory concentrations
MOMP	Mitochondria outer membrane permeability
DCFDA	2′,7′-Dichlorofluorescein diacetate
Baf	Bafilomycin A1
AO	Acridine orange
PE	Phosphatidylethanolamine
CQ	Chloroquine
3-MA	3-Methyladenine
Rapa	Rapamycin
CCK-8	Cell Counting Kit-8
PFA	Paraformaldehyde
EdU	5-Ethynyl-20-deoxyuridine
BSA	Bovine serum albumin
SDS-PAGE	Sodium dodecyl sulfate–polyacrylamide gel electrophoresis
ECL	Enhanced chemiluminescence
ANOVA	Analysis of variance
