# Supplementary material for: N-Methylparoxetine Blocked Autophagic Flux and Induced Apoptosis by Activating ROS-MAPK Pathway in Non-Small Cell Lung Cancer Cells
Source: Int J Mol Sci. 2019 Jul 11;20(14):3415. doi: 10.3390/ijms20143415 (PMC6678301; doi:10.3390/ijms20143415)
Supplement: Supplementary file 1 [file ijms-20-03415-s001.pdf]

Supplementary Materials:

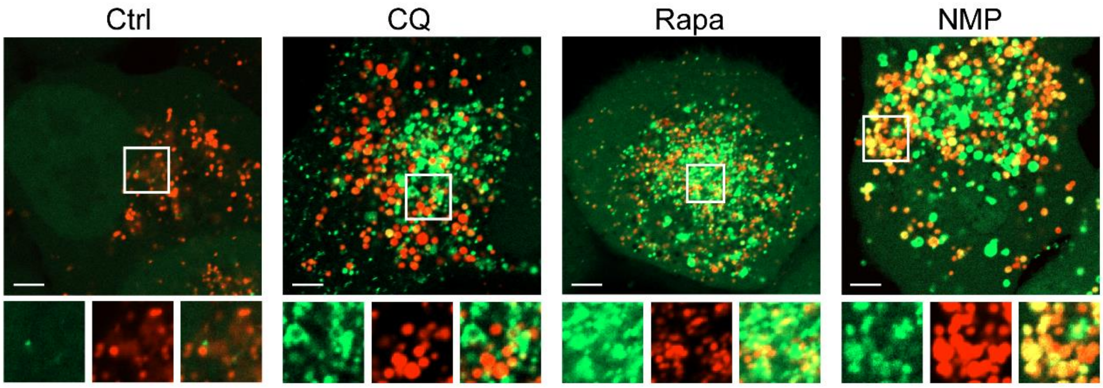

Figure S1. NMP failed to inhibit autophagosome-lysosome fusion. Fluorescence micrographs of GFP-LC3-expressing NSCLC cells labelled with LysoBrite™ Red (22645, AAT Bioquest, CA, USA). Cells were either treated with vehicle (ctrl), chloroquine (20 μM, CQ, an autophagosome-lysosome fusion inhibitor), rapamycin (1 μM, Rapa, an autophagy inducer) and NMP (40 μM). Red fluorescence, lysosome; green fluorescence, LC3-GFP; yellow fluorescence, merge of red and green signals, indicating fusion of autophagosome and lysosome. Scale bar, 5 μm.

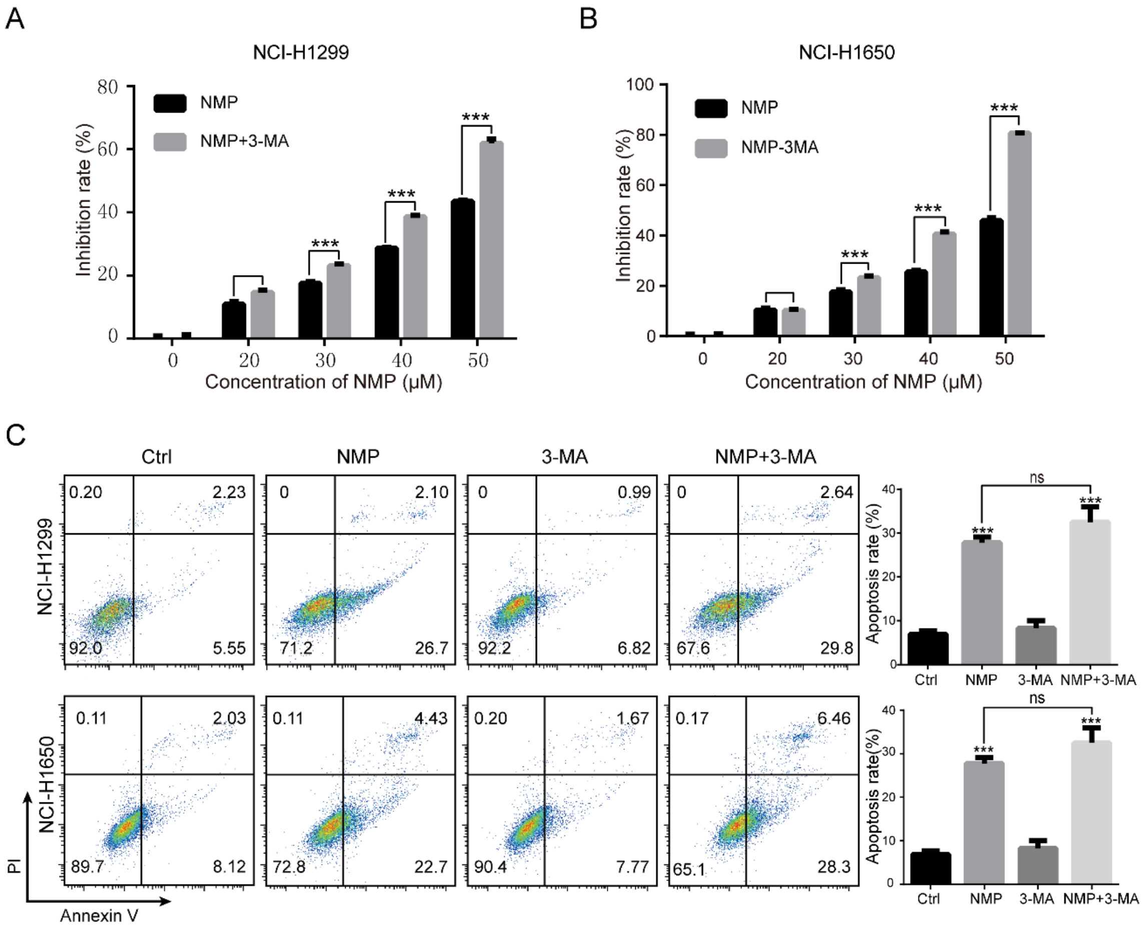

Figure S2. Blocking autophagosome formation failed to protect cells against NMP-induced cell death. (A, B) Cell viability of NCI-H1299 (A) and NCI-H1650 cells (B) treated with serial concentrations of NMP with or without 3-MA (5 mM) for 24 h. **(C)** Left, typical flow cytometry analyses of NCI-H1299 and NCI-H1650 cells treated with NMP and 3-MA (5 mM) alone or in combination for 24 h with Annexin V/PI staining (Left). Right, quantification of apoptosis rate from 3 independent flow cytometry experiments. Error bars, means ± S.D. of three independent experiments; ***$p < 0.001$, compared to the control group.
